# Erratum to “Impairment in S-phase entry of splenocytes of *Parp-1* knockout mice” [Proc. Jpn. Acad., Ser. B 79, No. 8, 248–251 (2003)]

**Published:** 2004-06-01

**Authors:** Fumiaki Watanabe, Mitsuko Masutani, Nobuo Kamada, Hiroshi Suzuki, Hitoshi Nakagama, Takashi Sugimura, Hirobumi Teraoka

In this paper, the phrase should be corrected as follows:

(page 249, right column, line 10)

For “with 50 μM ionomycin/1 μM phorbol”

read “with 0.5 μM ionomycin/10 nM phorbol”.

